# Androgen Receptor and Cardiovascular Disease: A Potential Risk for the Abuse of Supplements Containing Selective Androgen Receptor Modulators

**DOI:** 10.3390/nu15153330

**Published:** 2023-07-26

**Authors:** Ellis Hall, Misha F. Vrolijk

**Affiliations:** Department of Pharmacology and Toxicology, Maastricht University, 6229 ER Maastricht, The Netherlands

**Keywords:** androgen receptor, cardiovascular disease, SARMs, hypertension, atherosclerosis, myocardial hypertrophy, stroke, androgen receptor signaling

## Abstract

The androgen receptor (AR) is a member of the family of ligand-activated transcription factors. Selective androgen receptor modulators (SARMs) exert their biological function through complex interactions with the AR. It has been speculated that overexertion of AR signaling cascades as a result of SARM abuse can be a risk factor for the development of various cardiovascular diseases. The present literature review explores the implications of the interaction between SARMs and the AR on cardiovascular health by focusing on the AR structure, function, and mechanisms of action, as well as the current clinical literature on various SARMs. It is shown that SARMs may increase the risk of cardiovascular diseases through implications on the renin–angiotensin system, smooth muscle cells, sympathetic nervous system, lipid profile, inflammation, platelet activity, and various other factors. More research on this topic is necessary as SARM abuse is becoming increasingly common. There is a noticeable lack of clinical trials and literature on the relationship between SARMs, cardiovascular diseases, and the AR. Future in vivo and in vitro studies within this field are vital to understand the mechanisms that underpin these complex interactions and risk factors.

## 1. Introduction

Selective androgen receptor modulators (SARMs) are a class of synthetic, orally bioavailable compounds that exert their biological function through the androgen receptor (AR) as either agonists or antagonists [1,2,3]. SARMs have been touted to elicit selective anabolic effects of endogenous androgens through interactions with the androgen receptor (AR), supposedly with far less side effects than traditional androgen therapy [2,4]. As a result of this, SARMs containing supplements have gained popularity since their inception in the late 1990s within the fitness and bodybuilding world due to their muscle-building, fat-burning, and endurance-improving properties [1,5]. The social media prevalence of SARMs has been cited as a key aspect in the widespread use (and abuse) of SARMs in recent years. SARMs are often pushed as a ‘safer’ alternative to traditional androgenic anabolic steroids, while retaining the anabolic properties. Despite being illegal for human recreational use [1], SARMs are often marketed as research chemicals, allowing them to be bought online legally and easily [6]. The lack of regulation, in combination with a noticeable social media presence, has led to widespread abuse of these compounds by fitness enthusiasts [7,8].

SARMs have raised concerns about the implications that abuse may have on the health of those who take them. In particular, it has been suggested that the recreational use and abuse of SARMs may have negative implications on cardiovascular health and the development of cardiovascular diseases (CVDs) [1]. Recent evidence has suggested that SARM-induced CVDs are a very real risk for those who abuse SARMs [9]. The relative ease at which people can obtain and abuse SARMs, in combination with the potential negative implications on cardiovascular health, is a growing problem within the fitness community.

Due to the significant lack of clinical trials regarding SARMs and CVDs, the exact pathophysiology of how SARMs may lead to various CVDs is unclear. Since SARMs exert their biological activity through interactions with the AR, it is speculated that SARMs may lead to the development of CVDs through interactions with the AR at supra-physiological doses. Supra-physiological doses of androgens refers to when doses of androgens are above physiological levels, which results in increased or ‘exaggerated’ levels of AR signaling cascades as a result of the abundance of ligands for the AR [10]. This, for instance, occurs when individuals undergo androgen-replacement therapy, such TRT, in which supra-physiological doses of testosterone are injected intravenously [10,11]. Despite not being endogenous ligands, SARM abuse can also result in exaggerated AR signaling, essentially mimicking the effects of supra-physiological doses of endogenous AR ligands, hence the anabolic effects [1]. As such, SARMs may have similar associated risks if abused to the point where AR signaling cascades are upregulated to an exaggerated degree. The present review focuses on the interaction between SARMs and the AR and its implications for the risk of CVDs after recreational use of the SARMs.

## 2. Main Body

### 2.1. Androgen Receptor

The AR is a member of the steroid receptor family of ligand-activated transcription factors [12]. The AR is expressed in a diverse range of tissues, and it is involved in various biological processes in bone, muscle, prostate, adipose tissue, as well as the cardiovascular, immune, and reproductive systems [13]. This receptor is activated by a wide range of ligands, both exogenous and endogenous, including natural hormones, growth factors, peptides, as well as synthetic molecules, all known as androgens [12]. Endogenous androgens are the primary male sex steroids and are critical for the development of the male phenotype during embryogenesis, the development of sexual maturation during puberty, and maintenance of male reproductive function and behavior [14,15]. Exogenous androgens generally come in four basic types, including prescription testosterone-replacement therapy, anabolic steroids (e.g., trenbolone, oxandrolone), prohormones (e.g., androstenedione, dehydroepiandrosterone), and testosterone boosters (e.g., Tribulus terrestris, panax ginseng). In addition, there are other classes of exogenous androgens that do not fit neatly into these classes, such as selective androgen receptor modulators (SARMs), which are synthetic ligands of the androgen receptor. Exogenous androgens are used both clinically [16] and recreationally, most often by athletes [17].

#### AR Function

The primary endogenous androgens, such as testosterone and dihydrotestosterone (DHT), perform their function as ligands and exert their effects through complex interactions with the androgen receptor. The AR functions as a ligand-inducible transcription factor that mediates the expression of target genes [15,18]. As well as the well-documented AR genomic signaling pathway, the AR can also undergo a non-genomic signaling cascade [18,19]. These two mechanisms of AR action are further explained in Section 2.3 and Section 2.4, respectively. The general function of the AR is to regulate the expression of various genes that control the characteristics of male development, such as hair growth, skeletal muscle growth, sex drive, and the maintenance and development of male reproductive organs both before and after puberty [20]. The AR is however also involved in the pathophysiology of several diseases, such as prostate cancer and androgen insensitivity syndrome [20]. Despite the fact that the AR is considered to be pivotal to male development, it has also been suggested to play a role in female reproduction and the development of androgen-associated diseases [20].

### 2.2. AR Structure

**Figure 1:**
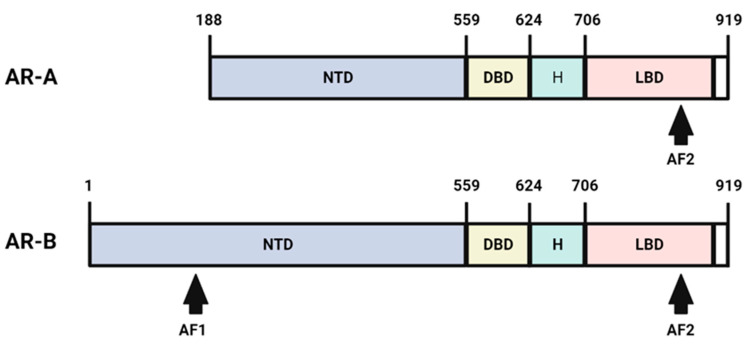
Structural domains of AR-A and AR-B.

The AR is a modular protein that is organized into various domains [14]. These include an *N*-terminal regulatory domain (NTD), a DNA-binding domain, a hinge region, and a ligand-binding domain (LBD) [14]. There exist two isoforms of the AR, namely AR-A and AR-B (87 kDa and 100 kDa, respectively). The structural domains of these isoforms are shown in Figure 1. Within the structural domains of the AR, multiple signal sequences are present [13]. As such, two primary transcriptional activation functions have been identified: the ligand-independent activation function 1 (AF1), located in the NTD; and the ligand-dependent activation function 2 (AF2), located in the LBD [13]. AF1 is required for maximal AR activity and mediates most of the transcription activity in the AR [13]. AF2 is responsible for the forming of co-regulatory binding sites and mediating direct interactions between the NTD and LBD [13].

#### 2.2.1. *N*-Terminal Regulatory Domain

Within the AR, the majority of the transcriptional activity is mediated by the NTD, which represents approximately half of the AR coding sequence [14]. Deletion of the LBD from the AR results in a *N*-terminal fragment that has very similar transcriptional activity to the activity of the full-length ligand-bound AR [21], suggesting that it contributes hugely to the transcriptional function of the AR. It has been suggested that the NTD within the AR is a flexible platform for the recruitment and assembly of co-regulators, as well as members of the transcriptional process [14]. The NTD acts as the primary mediator of the cell and gene-specific function of androgens [14]. There are differences between the AR-A and AR-B subtypes regarding these mediating functions, with AR-A lacking the ability to stimulate cell proliferation potentially due to the reduced binding of AR co-activating proteins to the NTD due to its smaller size [22]. The NTD and the LBD control transcription via AF1 [23].

#### 2.2.2. DNA-Binding Domain

The DBD contains a short *C*-terminal extension that forms part of the hinge, as well as two zinc finger motifs [14]. The two zinc finger motifs have differing functions [24,25]. The first zinc finger motif is responsible for the mediation of DNA recognition. This occurs through interactions with specific base pairs in response elements [25]. This facilitates the binding of the receptor with DNA [14]. The second zinc finger motif is responsible for the conservation of amino acids, which stabilize the DNA-bound receptor complex and mediate dimerization between steroid receptor monomers. This dimerization has been cited to stabilize the AR on DNA, which allows for additional bonds between homodimer members [14].

#### 2.2.3. Hinge Region

The H region exists between the DBD and the LBD [26]. It contains a bipartite nuclear localization signal and some necessary sites for phosphorylation, acetylation, and degradation [14]. Hence, the H region is involved in each of these processes within the context of the AR. The H region is proposed to play a lesser role in AR function when compared to the NTD, DBD, and LBD [26].

#### 2.2.4. Ligand-Binding Domain

The LBD is responsible for the high binding affinity of the AR to androgenic ligands [14]. The LBD contains a ligand-binding pocket composed of the ordered arrangement of 12 alpha helices [14]. The LBD controls transcription via AF2 [23]. The ligand-dependent AF2 is located in the LBD, and deletion of the AF2 domain dramatically reduces transcriptional activity in response to a ligand [14].

### 2.3. Genomic Mechanism of AR Action

**Figure 2:**
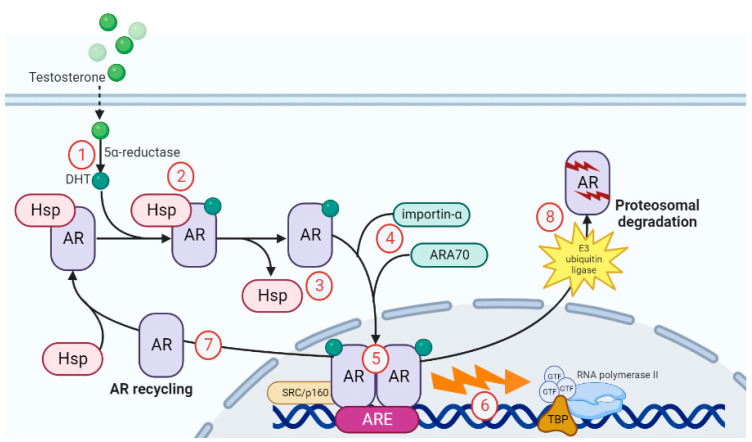
Genomic action of the AR consisting of eight different steps that ultimately lead to nuclear translocation of the AR, followed by the activation of gene transcription of androgen-responsive genes and the proteosomal breakdown of the AR. A detailed description of the pathway is given in Section 2.3.

Without a bound ligand, the AR is located primarily in the cytoplasm where it binds with heat-shock proteins (HSPs) and cytoskeletal proteins [19]. HSPs tether AR in the cytoplasm via the cytoskeletal proteins, such as filamin, which modulates AR confirmation in anticipation of efficient ligand binding. Genomic AR action is often referred to as the classical AR cycle and is illustrated in Figure 2. In the case of the primary endogenous ligand, genomic AR signaling begins with the conversion of testosterone into DHT via the 5α-reductase enzyme (1). DHT moves into the epithelial cell cytoplasm where it binds to the AR and forms a complex (2). This binding process induces a confirmational change, causing HSPs to disassociate from the AR (3). Other proteins, such as importin-α and ARA70, are recruited to stabilize the AR and promote AR translocation into the nucleus (4). The AR proceeds to dimerize within the nucleus (5). The genomic aspect of this cycle begins with numerous co-activators binding to the AR in the nucleus. The AR-DBD then facilitates nucleic acid binding at androgen response elements (AREs), which promotes the recruitment of co-activators with histone acetyltransferase activity via SRC/p160 co-activator family members, resulting in chromatin remodeling. This in turn allows the binding of TATA-binding proteins (TBPs), followed by GTF and RNA polymerase II to begin transcription (6). Non-ligand-bound AR elements may either be sent back to the cytoplasm and recycled for future ligand binding (7); alternatively, they may be targeted for proteasomal degradation after ubiquitination by E3 ubiquitin ligase (8). This degradation theory is part of the updated model genomic AR signaling. Previously, it was believed that non-ligand-bound AR was always exported from the nucleus after transcription (shown in step 7); however, it is now believed that instead of being exported, ligand-free AR in the nucleus is degraded (shown in step 8) [27].

### 2.4. Non-Genomic Mechanism of AR Action

**Figure 3:**
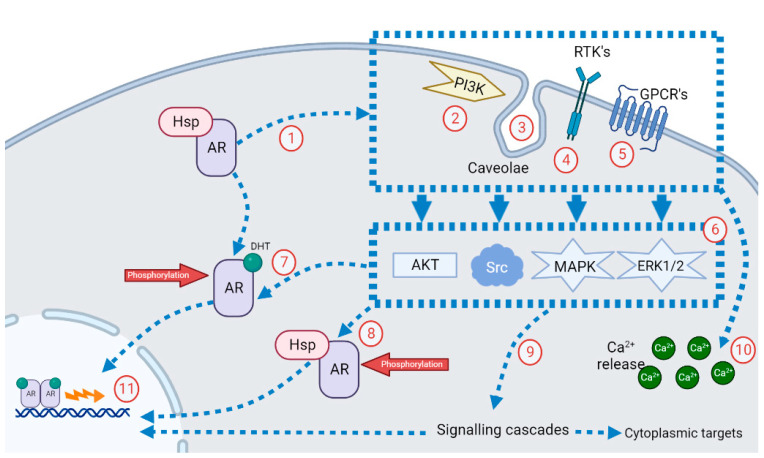
The non-genomic action of the AR consists of 11 steps, including the interaction of the AR with signaling molecules within the membrane of the cell, which starts a cascade of reactions that regulate separate cytoplasmic targets, as well as nuclear receptors and transcription factors within the cytoplasm. A detailed description of the pathway is given in Section 2.4.

Aside from the well-documented genomic signaling of the AR, evidence of a non-genomic AR signaling pathway has emerged within the last 20 years. Non-genomic AR signaling occurs within a case of seconds to minutes, implying a lack of transcription and translation from androgen-responsive genes [19]. The proposed mechanisms of non-genomic AR signaling are illustrated in Figure 3. The activated AR can interact with signaling molecules within the plasma membrane (1) and with various other structures, including the p85 regulatory subunit of PI3K (2), caveolae (3), receptor tyrosine kinases (RTKs) (4), and G-protein-coupled receptors (GPCRs) (5). Examples of signaling molecules activated by these structures include Akt, Src, MAPK, and ERK1/2, which can also phosphorylate the AR (6). Phosphorylation of ligand- (7) and non-ligand-bound (8) AR complexes by these compounds prevents AR degradation and is proposed to enhance AR nuclear translocation and activity. The majority of AR non-genomic signaling activity triggers signaling cascades (9) that regulate separate cytoplasmic targets, as well as nuclear receptors and transcription factors within the cytoplasm. The primary cytoplasmic target signaling event initiated is the release of intracellular Ca^2+^ release from the endoplasmic reticulum and/or mitochondria (10). It has been suggested that intracellular Ca^2+^ ions stimulate the binding of AR ligands to the AR [28].

As a result of these proposed mechanisms of AR non-genomic activity, it has been suggested that non-genomic signaling ultimately serves to influence the AR regarding its genomic activity (11), as well as other nuclear receptors [19]. Thus, the presence of this non-genomic signaling implies the existence of an autocrine positive feedback loop, whereby AR-activated kinases can phosphorylate the AR regardless of the presence of an AR ligand [19]. As mentioned, phosphoinositide 3-kinase, Akt, and ERK1/2 are all examples of kinases that can phosphorylate and activate the AR with and without an androgen, showing the adaptive nature of AR genomic activity in cellular environments with varying androgen levels [19].

### 2.5. Androgen Receptor Agonists and Antagonists

The AR most commonly interacts with endogenous agonistic ligands, such as T and DHT, in order to produce their biological effects [23]. However, besides endogenous compounds, exogenous compounds can also interact with the AR. These exogenous compounds can act as agonists or antagonists to the AR [23]. In the case of antagonists, these compounds are known as anti-androgens and have been shown to have positive implications on reducing the growth of cancer cells [29]. Examples of AR antagonistic pharmaceuticals include bicalutamide and hydroxyflutamide [23]. Exogenous AR agonists, however, are primarily used in clinical settings to treat diseases associated with low androgen receptor expression, such as hypogonadism [30]. In cases like these, TRT is often the most common approach, which works by injecting exogenous T to increase AR agonistic activity [30].

Exogenous AR agonists are also often used in sport and bodybuilding and have gained popularity in recent decades [31]. Androgenic anabolic steroids (AASs) are synthetic derivatives of testosterone that act as AR agonistic ligands and are often used for their hypertrophic and weight-loss characteristics, as well as increased libido and other benefits [31]. AASs are taken either orally (e.g., methandrostenolone, oxandrolone) or via injection (e.g., trenbolone, methenolone) [31]. Despite the benefits on AASs on those looking to attain the perfect body, they are known to have a multitude of side effects, including elevated blood pressure, decreased HDL, thrombosis, acne, gynecomastia, depression, and many more [31]. As a result of this, in recent decades, alternatives to AASs have emerged, which could retain the desired anabolic effects without the side effects [32]. One such example is the SARM class of synthetic AR ligands.

## 3. SARMs

### 3.1. SARMs as AR Ligands

SARMs are a novel class of tissue-selective AR ligands [4]. SARMs are often characterised by high specificity for the AR, high oral bioavailability, suitable pharmacokinetics for once-a-day administration, and tissue-selective pharmacological activities [4]. Despite sharing many similarities to AASs, SARMs are not technically anabolic steroids: they are synthetic ligands that bind to the AR [1]. They have been used both for clinical [3] and recreational use [1]. Some of the most popular SARMs on the market include ostarine, ligandrol, andarine, and testolone [1].

#### SARMs Mechanism of Action

**Figure 4:**
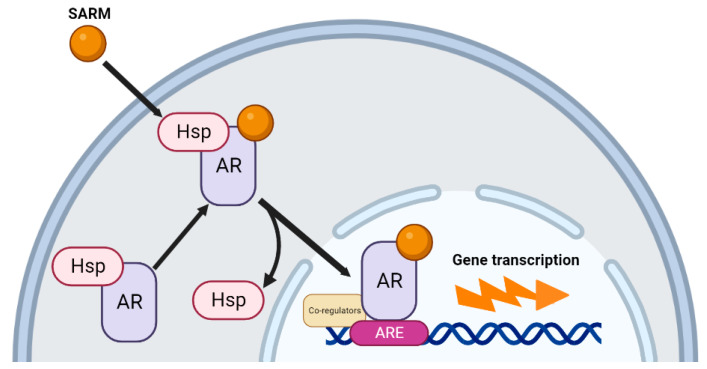
SARMs mechanism of action on the AR.

The mechanism of action of SARM signaling is similar to other endogenous androgens. The mechanism of SARM action is shown in Figure 4. SARMs enter the cytoplasm, displacing the AR from heat-shock proteins [3]. After binding to the AR, translocation to the nucleus occurs, where the complex acts as a transcription factor by binding to ARE. Co-regulatory proteins aid in determining and modulating the transcriptional response, based on the regulatory environment of the cell and the tissue type.

### 3.2. Clinical Use of SARMs

Companies such as Pfizer, Johnson and Johnson, Merck, and Glaxo began developing SARMs in the late 1990s due to preclinical evidence suggesting potent tissue selectivity and anabolic activity [12]. The major goal of the development of SARMs is to increase their tissue selectivity to avoid undesirable side effects [4]. This would significantly increase their clinical application in the treatment of various AR-related diseases [4]. The current therapeutic potential of SARMs lies in their potential to treat various diseases [3]. Depending on their chemical structure, SARMs can act as agonists, antagonists, partial agonists, or partial antagonists of the AR within differing tissues. As a result of this, SARMs have shown promise in the treatment of a variety of diseases, including osteoporosis [3], Alzheimer’s disease [3], cachexia [3], muscular dystrophy [3], sarcopenia [1], and hypogonadism, [33], as well as in male contraception [3]. For the most part, the role of the AR is significantly different from androgens regarding the development of various disease outcomes. The high selectivity and specificity of SARMs allow for AR activity to be increased without affecting endogenous androgen levels, which can be beneficial for alleviating low-androgen-related diseases. This may be a better strategy than the administration of androgens (as is the case with TRT), as this results in supra-physiological androgen levels, which has been shown to have physiological changes, such as worsened lipid profiles and hypertension, which can result in the development of various diseases [34]. As well as their clinical potential, SARMs have gained significant attention as performance-enhancing supplements in the previous two decades due to their lean-muscle-mass-building, fat-cutting, recovery, and endurance-increasing properties [1].

### 3.3. Recreational Use of SARMs as Sport Performance Enhancers

SARMs were banned in professional sports in 2008 by the World Anti-Doping Agency, and in 2017, the FDA issued a public advisory statement that products containing SARMs posed a high risk for harmful side effects [1]. SARMs are legal to use as research chemicals, but illegal for human consumption [1]. Despite this, SARMS still undergo widespread use within bodybuilding and sports. Some of the most popular SARMs on the market include ostarine, ligandrol, andarine, and testolone [1]. Here, we provide a review of the clinical literature on each of these SARMs as performance enhancers, as well as the associated risks.

#### 3.3.1. Ostarine

Ostarine (also known as enbosarm) is an orally bioavailable, nonsteroidal SARM that has been extensively studied for its potential in treating muscle-wasting conditions. It has shown positive effects on lean muscle mass and strength, as well as decreases in fat mass [1,35]. Common low-grade side effects of ostarine include headache, nausea, fatigue, and back pain. Other observed effects include increases in alanine transaminase and decreases in HDL, blood glucose, and insulin resistance, all of which returned to normal upon stopping ostarine treatment [1,35]. Information from bodybuilding forums and fitness enthusiasts cited 10 mg to 30 mg daily as the optimal dose for a minimum of 12 weeks, which is 10 times higher than the clinically studied dose, with anecdotal evidence suggesting that taking ostarine for much longer than this can suppress free T levels [1]. There also does not seem to be any known drug–drug interactions [36]; however, there exists some anecdotal reports mentioning SARMs as the best to take together or ‘stack’ [1] to improve their muscle-building and fat-loss properties.

#### 3.3.2. Ligandrol

Ligandrol is an orally bioavailable nonsteroidal SARM [1]. Ligandrol is less clinically explored than other SARMs such as ostarine. Ligandrol has been shown to have positive implications on muscle mass, with a reported slight increase in strength, but no statistically significant implications on fat reduction in the only clinical trial that has been performed on ligandrol as of yet [1,37]. Reported side effects include headache and dry-mouth; however, the drug seems to be well tolerated [1]. Physiological markers have been shown to change in a dose-dependent manner, with some biomarkers, such as follicle-stimulating hormone and free T, being significantly suppressed at higher doses but returning to normal upon drug cessation. In this clinical trial, despite clinical biomarkers of liver injury, such as aspartate aminotransferase (AST) and alanine aminotransferase (ALT) not being significantly altered at any dose [1], there exists clinical reports of ligandrol-induced liver injury [38,39], which makes it clear that more clinical trials are necessary on the implications of ligandrol on various biomarkers. Fitness forums have advised users to take between 5 mg and 10 mg daily for 10 weeks, with there being a reported risk of testosterone production upon increasing the dose past 10 mg per day. Similar to ostarine, ligandrol is often ‘stacked’ with other SARMs for improved benefit in terms of muscle building [1].

#### 3.3.3. Andarine

Andarine is an orally bioavailable SARM, which has no human clinical trials. Anecdotally, it has been touted as a muscle-boosting and fat-loss supplement [1]. Andarine has raised concerns primarily due to the fact that altered vision has been reported in various cases as a result of andarine ingestion [1,6,40]. Suppression of testosterone is another associated risk [1]. Andarine is reported to be weaker than other SARMs, so it is often ‘stacked’ with other SARMs, such as testolone, ligandrol, or cardarine [1]. Slight improvements in muscle building and fat burning are purportedly noticeable above 50 mg daily for more than 12 weeks, with the dose able to be lowered when stacking with other SARMs. Low doses of andarine (25 mg) can anecdotally have implications on improved mood but a negligible impact on fitness parameters [1].

#### 3.3.4. Testolone

Testolone is an orally bioavailable nonsteroidal SARM [41] developed by Radius health, Inc [1]. As is the case with most SARMs, safety is unclear, and results are mostly anecdotal. It has been suggested that testolone has an acceptable safety profile in a recent cancer study [42], and anecdotal evidence suggests increases in lean muscle mass and strength [1] and decreases in fat mass [43]. In a recent study in mice, however, it has been reported that testolone did not significantly increase skeletal muscle but did increase liver injury biomarkers [44]. This same study mentioned the possibility that RAD140 may act as an agonist for the AR outside of the muscles since RAD 140 supplementation decreased the effects of testosterone on the prostate and seminal vesicles [44]. There is evidence of testolone-induced liver injury [38,45], which is in line with reported increases in liver injury biomarkers, such as ALT, AST, and general liver inflammation [1], all of which returned to normal following 12 months of drug cessation. Testolone may also have side effects, such as sleepiness and lethargy. Fitness forums recommend 5 mg to 30 mg daily dose for 16 weeks [1].

## 4. The AR and Cardiovascular Events

### 4.1. AR and CVD

CVDs are the highest leading cause of death worldwide. Risk factors for cardiovascular disease are well documented in the scientific literature, including smoking, diabetes, hyperlipidemia, and gender, among others [34]. There is evidence that androgens can be both protective and detrimental to cardiovascular health [34,46]. The use of androgen therapies to treat CVDs is inconclusive, and so in some cases, focus has shifted away from androgens and onto the AR as a site for treating CVDs [34].

Targeting the AR instead of using androgens as a therapy method for CVDs poses multiple benefits. Firstly, targeting the AR will result in far less side effects, as it will not affect whole-body androgen levels and will work much more locally [34]. Secondly, targeting the AR will likely affect body lipid profiles to a far smaller degree than androgen therapy, which can have implications on some CVDs [34]. Thirdly, the AR can be targeted in selective cell types, rather than the whole body [34]. Fourth, non-androgen-mediated AR activity can be targeted, which is not possible with traditional androgen therapy. There is evidence that use of AR-degradation enhancers (such as ASC-J9^®^) could be used to target CVDs without affecting androgen levels and resulting in unwanted side effects [34], but on the other hand, this poses the question: what are the implications of high AR activity on the incidence of CVDs under supra-physiological androgen doses? As a preface, there is evidence that low androgen levels can promote CVDs [47,48] and that androgens at normal physiological levels can have a protective effect on the myocardium and cardiovascular system in general [49]. The following sections focus on how excessively high or supra-physiological androgen levels (and as a result, high amounts of AR signaling) can have implications on the incidence of various CVDs, since it applies to the case of SARM abuse as a class of exogenous androgen receptor ligands.

#### 4.1.1. Hypertension

Hypertension is one of the most prevalent CVDs, affecting 25% of the US population [34]. There are several risk factors for hypertension, including age, obesity, alcohol abuse, and gender differences. A key aspect of hypertension is that it is a preliminary factor in the development of further CVDs, such as stoke and atherosclerosis [34]. Interestingly, men generally have higher blood pressure than women, but this difference is lost after menopause in women, and men have declining androgen levels past age 70. As a result of these findings, there has been investigations into the effect of androgen levels in the development of hypertension due to the clear differences between the sexes throughout their lifespan. Investigating the relationship between androgens/AR and hypertension can be beneficial in the future treatment of this and other CVDs.

There are multiple mechanisms by which AR activity can have implications on hypertension. Firstly, androgens have been shown to increase renin levels and expression of angiotensin-converting enzyme (ACE) and angiotensin II receptor type 1 (AT1R), while simultaneously decreasing angiotensin II receptor type 2 (AT2R) [50]. AT1R has a vasoconstriction effect, whilst AT2R has a vasodilatory effect, and these receptors are seen to counterbalance each other [51]. These factors together favor a vasoconstrictor pathway, increasing angiotensin II (Ang II) activity and enhancing Ang II vascular responsiveness to androgens [50].

Ang II-induced vascular constriction is seemingly important in the development of hypertension. In line with this, in various rat studies, androgen deficiency via castration decreases blood pressure through decreased Ang II activity [52], and conversely, exaggerated androgen levels have been shown in Ang II-induced hypertension [50]. Additionally, high androgen levels have been shown to result in increased aldosterone stimulation [53], stimulating sodium retention and increasing the risk of hypertension [54]. These findings all together provide evidence that high AR activity and high androgen levels may have implications for hypertension via alterations in the renin–angiotensin–aldosterone system (RAAS) [50,54].

**Figure 5:**
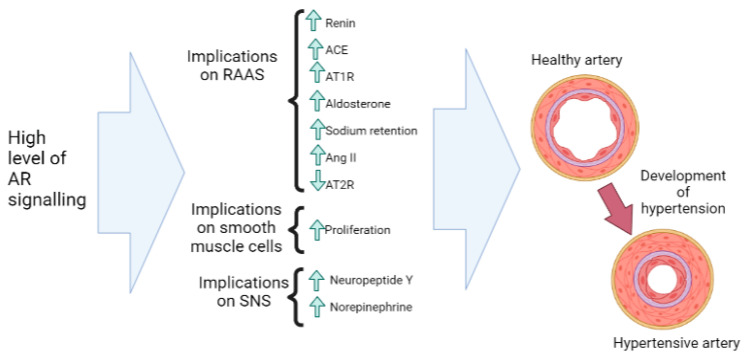
The potential effects of high levels of AR signaling on the risk of hypertension. There is evidence that AR signaling increases renin, ACE, AT1R, and aldosterone (hence increasing sodium retention) and decreases AT2R, with implications for the RAAS, resulting in Ang II-induced hypertension. As well as this, AR signaling may have implications on the SNS, increasing neuropeptide Y and norepinephrine levels, as well as increased smooth-muscle-cell proliferation, leading to hypertension. ACE: angiotensin-converting enzyme, Ang II: angiotensin II, AR: androgen receptor, AT1R: angiotensin 2 receptor type 1, AT2R: angiotensin 2 receptor type 2, RAAS: renin–angiotensin–aldosterone system, SNS: sympathetic nervous sytem.

As well as the implications of AR activity on the RAAS, there is also potential for AR activity to interact with the sympathetic nervous system (SNS). Androgens can increase neuropeptide Y release and increase norepinephrine synthesis [54], both of which contribute to autonomic circulation control [55]. This upregulation of neuropeptide Y and norepinephrine, as well as increased sympathetic nervous system activity, can contribute to vasoconstriction, which eventually may lead to hypertension [56]. There is also evidence that androgens and high AR activity can lead to increased smooth-muscle-cell proliferation, which can contribute to thickened arterial walls, further contributing to the risk of hypertension [54]. In summary, high AR signaling seemingly worsens hypertension. The potential effects of high levels of AR signaling on the risk of hypertension are shown in Figure 5.

#### 4.1.2. Atherosclerosis

Similar to hypertension, gender differences exist regarding the prevalence of atherosclerotic development [34]. Atherosclerosis is an immunoinflammatory disease of medium- and large-sized arteries fueled by lipids [57]. The most devastating consequences of atherosclerosis, such as heart attack and stroke, are caused by superimposed thrombosis [57]. Due to gender differences in the incidence of atherosclerosis between males and females, investigation into the effect of supra-physiological doses of androgens and AR signaling on atherosclerotic development has been conducted. The first mechanism by which AR signaling can lead to increased LDL and decreased HDL. There is evidence that supra-physiological doses of androgens and hence exaggerated AR signaling can contribute to increases in LDL and decreases in HDL [58]. It is important to note that this only applies to supra-physiological doses of androgens, as seemingly androgen therapy in hypogonadal men does not result in significant changes in LDL or HDL [58], possibly due to the absence to exaggerated or above physiological levels of AR signaling. These changes in lipid profiles can be proatherogenic, triggering an inflammatory response, which can lead to the formation of plaques in the arteries [59].

As well as an altered lipid profile, androgens and AR signaling can stimulate smooth-muscle-cell proliferation in the arterial walls [54]. This process is known as intimal hyperplasia and is considered to be a precursor for atherosclerosis in humans [60]. Additionally, human observation studies in athletes and patients have provided evidence that supra-physiological doses of androgens and hence exaggerated levels of AR signaling can induce enhanced platelet activity and thrombopoiesis [61]. This can lead to increased platelet aggregation [61], which can eventually contribute to the formation of blood clots that can block arteries. Another way that AR signaling may contribute to atherosclerosis is through the potential formation of pro-inflammatory cytokines, such as IL-1α, which may contribute to the formation of plaques [34] under supra-physiological androgen doses.

**Figure 6:**
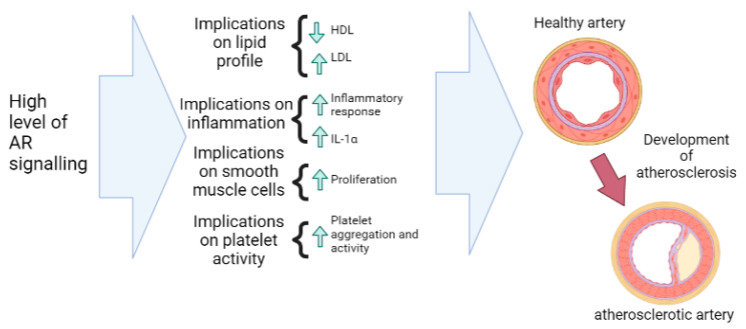
Potential effects of high levels of AR signaling on the risk of atherosclerosis. The specific factors include changes in blood lipid profiles (HDL, LDL) in the body, increased inflammation (IL-1α) in endothelial cells, increased muscle cell proliferation, as well as changes in platelet activity. ERK: extracellular signal-regulated kinase; LTCCs: L-type calcium channels; mTOR: mammalian target of rapamycin, SERCA: sarcoplasmic reticulum calcium ATPase.

The implications of high AR signaling under supra-physiological androgen levels on atherosclerosis are illustrated in Figure 6.

#### 4.1.3. Myocardial Hypertrophy

Myocardial hypertrophy (MH) is defined by the augmentation of ventricular mass as a result of increased cardiomyocyte size [62] and is a risk factor for heart failure and sudden cardiac arrest [34]. As is the case with various other CVDs, there are very clear gender differences when it comes to the prevalence of MH in men and women [34,63,64]. There exists evidence that treatment with estrogens may play a protective role in MH response, whilst androgens can result in increased MH [34,64], possibility due to higher levels of AR signaling and gene transcription. Estrogens have been shown to have anti-proliferative effects on cardiac fibroblasts and smooth muscle cells, whereas androgens increase proliferation of vascular smooth muscle cells [34]. Additionally, androgens may inhibit cardiac muscle degradation [65], most likely to a far greater degree under supra-physiological doses.

One mechanism by which androgens can promote MH is through the activation of the mammalian target of rapamycin (mTOR) signaling pathway [11]. Activation of the mTOR pathway can lead to an increase in the size and mass of individual muscle cells within the heart, contributing to hypertrophy [11]. Additionally, androgens can stimulate the proliferation of cardiac muscle cells through the activation of the extracellular signal-regulated kinase (ERK) signaling pathway [11], likely due to high levels of AR signaling under supra-physiological doses of androgens. This can lead to an increase in the number of cardiac cells in the heart and contribute to overall cardiac hypertrophy [11].

Androgens have also been shown to have implications on calcium homeostasis in cardiac muscle cells [46]. Here, androgens have been shown to upregulate the expression of L-type calcium channels (LTCCs) [46,66] and the sarcoplasmic reticulum calcium ATPase (SERCA) [67], which have implications on activating pathways, which contribute to cardiac hypertrophy [68]. The AR is very prevalent in myocardial tissues, resulting in significant levels of AR signaling, modulating the cardiac phenotype and producing hypertrophy by direct receptor-specific mechanisms involved in the modulation of many genes [34].

**Figure 7:**
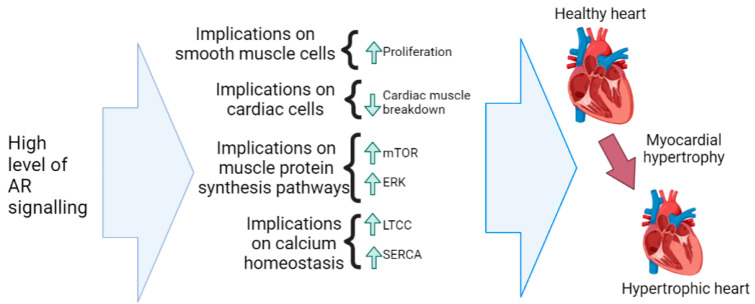
Potential effects of high levels of AR signaling on the risk of myocardial hypertrophy. The specific involved mechanisms are around increased muscle cell proliferation and decreased breakdown, as well as increases in muscle protein synthesis pathways (mTOR, ERK) and changes in calcium homeostasis (LTCC, SERCA) within cardiac muscle cells.

In summary, men have increased hypertrophy compared to age-matched women, with androgens likely promoting cardiac hypertrophy. This is most likely due to exaggerated levels of AR signaling. The implications of high AR signaling under supra-physiological androgen levels on myocardial hypertrophy are illustrated in Figure 7.

#### 4.1.4. Stroke

Stroke refers to a rapid loss of brain function due to ischemia caused by a blockage or hemorrhage of blood vessels [34]. The cerebral vasculature is a target for androgen action due to its high levels of sex steroid receptors (including the AR) and relevant metabolizing enzymes [69]. Risk factors include atherosclerosis, diabetes, heart valve defects, hypertension, and gender. Male stroke incidence is 33% and prevalence is 41% higher than female incidence [34]. This difference has been investigated as the AR seemingly has implications on stroke incidence. Like other CVDs, the effect of AR signaling on stroke incidence is paradoxical. Seemingly, both too low and too high levels of AR signaling can have implications on the risk of stroke. There is evidence that low endogenous AR levels can contribute to higher incidence of stroke and that androgen treatment worsens stroke outcomes under supra-physiological androgen doses [34]. AR signaling likely plays a major role in this difference. The inconsistencies of stroke incidence depending on various factors has led to the proposal that the AR plays different roles in individual cells, thus affecting stroke in various manners [34]. There are various mechanisms by which high levels of AR signaling can contribute potentially to stroke.

**Figure 8:**
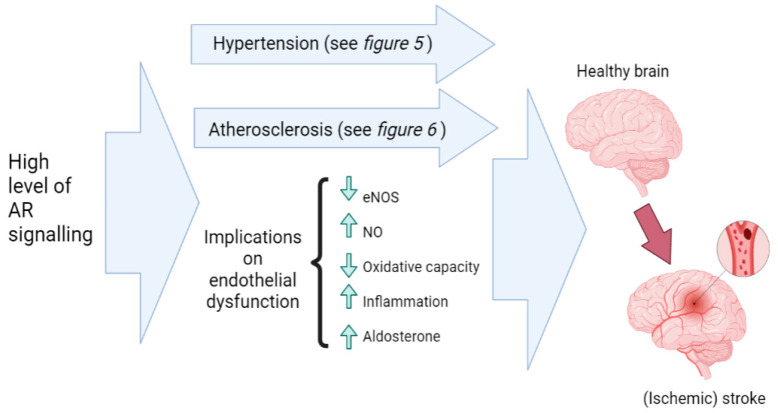
Potential effects of high levels of AR signaling on the risk of stroke. Hypertension and atherosclerosis are the main factors that cause stroke, but there is also evidence that endothelial dysfunction (altered eNOS, NO, oxidative capacity, inflammation and aldosterone) can contribute to stroke development [65], with there being evidence that high levels of AR signaling can lead to endothelial dysfunction. eNOS: endothelial NO synthase; NO: nitric oxide.

Some of the primary mechanisms that can lead to the development of stroke are other CVDs. Hypertension and atherosclerosis are known to cause stroke [70]. A stroke is the most devastating manifestation of these two other CVDs, with hypertension being the most significant risk factor in the development of stroke as it precedes atherosclerosis and stroke [70]. As mentioned in previous sections, there are many proposed mechanisms by which high levels of AR signaling can lead to the development of hypertension and atherosclerosis, including the implications of high AR signaling on RAAS, smooth muscle cells, SNS, blood lipid profile, inflammation, and platelet activity (see Figure 7 and Figure 8). As well as hypertension and atherosclerosis, it is also possible that high levels of AR signaling can lead to endothelial dysfunction, another potential risk factor for the development of stroke [71]. It has been shown that supra-physiological doses of T showed inhibition of the endothelial NO synthase (eNOS) gene, hence decreasing nitric oxide (NO) levels [72]. NO is a potent vasodilator that helps to maintain blood vessel health and function. In the same study, supra-physiological T doses also significantly decreased oxidative capacity and increased inflammation [72]. These factors suggest that supra-physiological androgen doses can lead to endothelial dysfunction [72], potentially due to exaggerated AR signaling activity. It is also possible that the increase in aldosterone seen during high AR signaling can contribute to endothelial dysfunction [73].

In summary, the potential implications of high AR signaling on the risk of stroke are illustrated in Figure 8.

## 5. SARMs and the Potential Risk for CVDs

### 5.1. SARMs and CVD

While the relationship between the AR/androgens and cardiovascular disease is far from conclusive, the risks associated with androgens and CVD are of real concern regarding the prevention of a cardiac event. In the case of SARMs, the relationship between (specifically exogenous) androgens/AR and CVDs is of great importance since SARMs exert their physiological effects through the AR. Improving our understanding of the cells in cardiovascular systems that are influenced by AR agonists will help to develop SARMs that have beneficial effects on cardiovascular health [74]. SARMs have a significant advantage over traditional androgen therapy in that they can be manufactured for better selectivity and potency at the AR and minimize side effects, including the risk of CVDs [74]. As a result of this, SARMs have the very real potential to treat those with low endogenous androgen levels, which as a clinical diagnosis has been shown to result in increased risk of CVDs [34]. Despite this, in line with the clinical literature, there is also a possibility that the use of SARMs can contribute to increased risk for CVDs as high levels of AR signaling can also contribute to increased risk of CVDs, as explained in chapter 3 [11,50,51,52,55,56,61,62,65,68]. It is also worth noting that whilst the majority of studies report a decrease in cardiovascular events in the long term as a result of increased physical activity, there is evidence that vigorous physical activity can increase the risk of sudden cardiac death and acute myocardial infarction [75]. The most common group of people who take SARMs are fitness enthusiasts and bodybuilders who engage in recreational or professional sport/bodybuilding [1]. In combination with the fact that the relative risk of sudden cardiac events has been reported to be elevated during and up to 1 h after exercise [75], it is likely that SARM abuse, alongside vigorous physical activity or weightlifting, is a risk factor for the development and/or incidence of (acute) CVDs. Additionally, genetic polymorphisms may affect the risk of androgen-related CVDs. Genetic polymorphisms of AR gene have been demonstrated to alter the efficacy of androgens and may also affect disposition and efficacy of androgens [18]. These polymorphisms have been recognized with endogenous androgens (such as T) to affect not only the efficacy of the AR and related genes, but also genes related to T disposition, such as SHBG and PDE7B [18], which may affect the removal of androgens, since expression of these genes is seemingly upregulated under supra-physiological concentrations of androgenic drugs [76]. As such, genetic polymorphism of the AR gene may serve as a useful genetic marker(s) for the assessment of cardiovascular risk [18].

### 5.2. Alternative Pathways

From a molecular perspective, are well as the receptor interactions (see Section 4), AR signaling pathways also consist of post-receptor and pre-receptor interactions. It is of significant interest to research how SARMs interact with the AR at different points in the signaling pathway(s), as more information about how SARMs interact with the process holistically is essential for the future development and clinical optimization of SARM, as well as risk reduction in the long term. Post-receptor interactions between SARMs are the AR are not well understood, but it is important to acknowledge that SARMs may give rise to different patterns of post-receptor signaling [77]. If it is found that SARMs can have implications for AR degradation, translocation, nucleus binding, etc., this may provide a discussion point for an alternative mechanism as to how SARM abuse can link to the development of CVDs.

Regarding the pre-receptor aspect of the AR signaling process, this refers to the interactions that occur before ligand binding to the AR. Androgens are synthesized by steroidogenic enzymes, which convert cholesterol into androgens [78]. Steroidogenic enzymes are involved in the biosynthesis and metabolism of steroid hormones, including androgens. These enzymes play a crucial role in pre-receptor AR interactions through the conversion of precursor molecules into active androgens, according to the pathology of steroidogenesis. Cholesterol side-chain cleavage enzyme (CYP11A1) initiates the conversion of cholesterol to pregnenolone, a precursor for all steroid hormones. 3β-Hydroxysteroid dehydrogenase (HSD3B) catalyzes the conversion of pregnenolone to progesterone. 17α-Hydroxylase/C17,20 lyase (CYP17A1) plays a dual role in the biosynthesis of androgens. It converts progesterone to either 17α-hydroxyprogesterone or 17α-hydroxypregnenolone and converts 17α-hydroxypregnenolone to dehydroepiandrosterone (DHEA) [78]. 3β-Hydroxysteroid dehydrogenase type 2 (3β-HSD2) converts DHEA into androstenedione, another precursor for T synthesis. 17β-hydroxysteroid dehydrogenase (HSD17B) converts androstenedione to testosterone. 5α-reductase converts T into DHT, a more potent androgen.

Determining whether SARMs can interfere with any key enzymes in the steroidogenesis pathway may be critical for understanding whether SARM abuse may lead to indirect cardiovascular outcomes. This could include low endogenous androgen-related CVDs [47,48], which may be a biproduct of altered pre-receptor steroidogenesis enzyme interactions since endogenous androgen levels may decrease due to these pre-receptor interactions. Whilst there is evidence that AAS abuse may interfere with steroidogenic genes or enzymes, it is unclear whether SARMs interact with these genes in the same way [79]. As it currently stands, there is evidence that various SARMs, including ligandrol and ostarine, do not affect steroidogenic enzymes/genes (such as CYP17A1) levels, and the implications of SARM abuse on other steroidogenic factors, such as the 5α- reductase enzyme, is unclear. As it stands currently, despite anecdotal reports of SARM abuse lowering endogenous androgen levels [1], the evidence that SARMs interact with steroidogenesis at a pre-receptor level is negligible. This represents a key point to investigate in the future because if SARMs interact with the pre-receptor stage of the AR signaling process, it could affect endogenous androgen levels and lead to negative health outcomes, both cardiovascular and elsewhere.

#### The Hypothalamic–Pituitary–Gonadal Axis

The hypothalamic–pituitary–gonadal axis (HPGA) is a critical hormonal system that regulates the production and release of sex hormones, including testosterone and estrogen, in the body. It involves a series of interactions between the hypothalamus, pituitary gland, and gonads [80]. The hypothalamus is a region in the brain that releases gonadotropin-releasing hormone (GnRH) in a pulsatile manner. GnRH acts as a signaling molecule that stimulates the next component of the HPGA. The pituitary gland, also located in the brain, responds to GnRH by releasing two gonadotropins—luteinizing hormone (LH) and follicle-stimulating hormone (FSH). LH and FSH travel through the bloodstream to the gonads [80]. In response to LH and FSH, the gonads produce and release sex hormones. In males, LH stimulates the Leydig cells in the testes to produce testosterone, while FSH promotes spermatogenesis. The HPGA plays a vital role in regulating fertility, sexual development, and reproductive functions in both males and females. It operates through a feedback mechanism, where sex hormone levels provide feedback to the hypothalamus and pituitary, modulating the release of GnRH, LH, and FSH [80].

There is evidence that SARM use can result in alterations of the plasma levels of anabolic hormones involved in the HPGA [81]. The effect of SARMs on the HPGA resemble that of exogenous T use regarding physiological alterations but to a lesser extent. Abuse or excessive use of SARMs may disrupt the HPGA by causing hormonal imbalances, which can lead to higher-than-normal levels of androgens in the body. These elevated androgen levels can trigger negative feedback on the HPGA, signaling the hypothalamus and pituitary to reduce the production of GnRH, LH, and FSH. As a result, endogenous T production may be suppressed or shut down [82]. A study with ligandrol in healthy men (21–50 years) showed dose-related suppression in total T and sex-hormone-binding globulin levels after 21 days of administration. However, the levels returned to baseline after 56 days of discontinuation of treatment [37,81]. It is possible that different SARMs affect the HPGA in different ways since there is evidence that in some cases, the HPGA profile is unaffected by SARM (enbosarm) use in both males and females, resulting in unchanged serum concentrations of free T, LH, and FSH [82,83]. However, at higher concentrations, it resulted in reduced sex-hormone-binding globulin (SHBG) and free T, once again suggesting dose dependence [82]. The differences between different SARMs at different doses makes it difficult to narrow down the effect that SARMs have on the HPGA (Machek et al., 2020); however, it seems highly likely that SARM use can suppress hormones involved in the HPGA. It also seems highly likely that at higher doses in situations where SARMs are being abused, the effect is exacerbated, and free T has been shown to be suppressed [82].

The implications of the relationship between SARM abuse and the HGPA on the development of CVDs lie within the increased likelihood of CVD development as a result of low endogenous androgen levels after ending an SARM cycle [34]. Prolonged use of SARMs without proper post-cycle therapy (PCT) can lead to altered lipid metabolism, changes in cholesterol levels (development of atherosclerosis), and other cardiovascular risk factors [34]. Testosterone has important cardiovascular effects in maintaining vascular health, and suppression of endogenous testosterone may lead to adverse changes in cardiovascular function, potentially increasing the risk CVDs. The interactions between SARMs and the HPGA may also represent a critical time during the male lifespan, whereby individuals may be most susceptible to the cardiovascular risks of SARM abuse. With HPGA activity and endogenous T levels being the highest from the late teens until the early 30s [84], this may represent an age range whereby SARMs may be the most disruptive to the highly active and volatile HPGA, suppressing T in the body. This phenomenon is widely explored with traditional AAS, and it is generally accepted that HPGA dysfunction can be derived from excessively high androgens, resulting in suppression of HPGA-related hormones [85,86]. Whilst SARMs may represent a therapeutic option to treat age-related hypogonadism later in life to avoid low-androgen-related CVDs, it is highly likely that SARMs interact with the HPGA; therefore the abuse of SARMs, especially for males in the 18–30 age range when the HPGA is most active, may suppress endogenous androgens, potentially leading to cardiovascular complications without sufficient PCT. The effect of different SARMs and doses on specific aspects of the HPGA requires more investigation. Despite these concerns, T suppression as a result of SARM use is often reversible [77].

### 5.3. The Duality of SARMs on CVDs

The development and use of SARMs is a double-edged sword. SARMs can be used to treat low-androgen-related diseases (such as hypogonadism or CVD induced by a lack of cardioprotective androgens), but if abused, it could contribute to an increased risk of certain CVDs. It is possible that SARM abuse can directly contribute to cardiac events, despite evidence being scarce. Padappayil et al. presented a case in 2022 of a healthy young male patient who presented with shortness of breath after self-medicating himself with the SARM testolone/RAD-140 for the purpose of performance enhancement and muscle hypertrophy [9]. The resulting clinical diagnosis of this case was SARM testolone (RAD-140)-induced acute myocarditis [9]. Cases such as these provide the possibility that abuse or self-medication of SARMs may result in CVDs, despite the direct mechanisms not being fully understood. If this is the case, there may be grounds for further investigation between SARMs and CVDs as a result of high levels of AR signaling.

## 6. Discussion

Overall, it is highly likely that SARMs contribute to the development of CVDs through interactions with AR. Whilst the exact mechanisms of how SARMs can lead to the development of CVDs is not known, the present literature review has provided some potential mechanisms through which SARMs may contribute to the development of various CVDs. It is also likely that the reason this occurs is as a result of an over-expression of AR signaling pathways. Similar to supra-physiological doses of androgens, a portion of the risk of cardiovascular health comes from the disruption of normal physiological homeostasis, with various signaling pathways being overexpressed, which can lead to the development of CVDs. More research is needed on how exactly this occurs in the case of SARMs and which specific AR-derived pathways can lead to worsened cardiovascular health, but it is highly likely that the risk of SARM abuse on CVDs is mediated by the AR and relevant gene cascades. In summary, this review finds that the following mechanisms that are potential contributing factors to the development of various CVDs are a result of exaggerated AR signaling. Regarding hypertension, high AR signaling may have implications on the RAAS, smooth muscle cells, and the SNS. Regarding atherosclerosis, high levels of AR signaling can lead to changes in blood lipid profiles in the body, increased inflammation in endothelial cells, increased muscle cell proliferation, and changes in platelet activity. Regarding MH, exaggerated AR signaling may lead to increased muscle cell proliferation and decreased breakdown, as well as increases in muscle protein synthesis pathways and changes in calcium homeostasis within cardiac muscle cells. Regarding the incidence of stroke, hypertension and atherosclerosis are the main factors that cause stroke, but there is also evidence that endothelial dysfunction can contribute to stroke development, with there being evidence that high levels of AR signaling can lead to endothelial dysfunction.

Research within the field of SARMs in relation to (cardiovascular) health is essential for a reduction in SARM abuse in the future. This is of great societal relevance since SARM abuse has been increasing in recent years due to a multitude of factors, as mentioned within the body of the present review. Currently, rules and regulations regarding SARM use are very relaxed, with many loopholes being present, allowing people to purchase SARMs with very little difficulty. The looseness of terms such as ‘research chemicals’ allow these products to be sold for research purposes, but it is very clear that this is not the case in most situations, with SARMs often being used recreationally as anabolic agents. The lack of a need to prove intentions for the use of the SARMs (in most cases), the lack of regulations regarding the purity of compounds, and the ease at which people can buy them, in combination with the stigma that SARMs are a safer alternative to traditional AAS, are some of the reasons as to why abuse is increasing. SARMs have great potential to be used to treat various diseases within a clinical setting, and SARM development should continue with this idea at the forefront. It is probable that SARMs are less likely to result in the development of CVDs or other ailments, such as liver failure, when compared to other anabolic agents, such as T or AASs, because SARMs generally having lower potency and higher selectivity and specificity for the AR. However, it is important to consider the resulting physiological changes that derive from AR signaling pathways being over-expressed.

It is very apparent that more in vivo and in vitro research is needed in order to better understand how individual SARMs may have implications on individual CVDs and to address the multitude of knowledge gaps within this field. More empirical research is critical for future development and knowledge expansion since the relationship between the AR, CVDs, and SARMs is still unclear. The AR response to compounds is pivotal to understanding their potency and, as such, is useful for understand the risks of using SARMs.

## Data Availability

No new data was generated in this study.
